# The impact of vitamin E, vitamin B6, and niacin intake on cataract incidence based on NHANES 2005-2008 data

**DOI:** 10.3389/fnut.2024.1406147

**Published:** 2024-08-09

**Authors:** Guo-Bin Zhuang, Xiang Li, Shi-Nan Wu, Si-Qi Zhang, Zhi-Jie Zhang, Nuo Dong

**Affiliations:** ^1^Department of Ophthalmology, Quanzhou First Hospital Affiliated to Fujian Medical University, Quanzhou, Fujian, China; ^2^Eye Institute and Affiliated Xiamen Eye Center, School of Medicine, Xiamen University, Xiamen, Fujian, China; ^3^Fujian Provincial Key Laboratory of Ophthalmology and Visual Science, School of Medicine, Xiamen University, Xiamen, Fujian, China; ^4^Huaxia Eye Hospital of Quanzhou, Quanzhou, Fujian, China; ^5^Department of Oncology, Xiang’an Hospital of Xiamen University, Xiamen, Fujian, China; ^6^Department of Ophthalmology, The First Affiliated Hospital of Kunming Medical University, Kunming, Yunnan, China; ^7^Xiamen Clinical Research Center for Eye Diseases, Xiamen, Fujian, China; ^8^Xiamen Key Laboratory of Ophthalmology, Xiamen, Fujian, China; ^9^Fujian Provincial Key Laboratory of Ocular Surface and Corneal Disease, Xiamen, Fujian, China; ^10^Xiamen Key Laboratory of Corneal and Ocular Surface Diseases, Xiamen, Fujian, China; ^11^Translational Medicine Institute of Xiamen Eye Center of Xiamen University, Xiamen, Fujian, China; ^12^Department of Ophthalmology, Affiliated People’s Hospital and Zhenjiang Kangfu Eye Hospital, Zhenjiang College, Zhenjiang, Jiangsu, China

**Keywords:** vitamin E, vitamin B6, niacin, cataract prevention, NHANES

## Abstract

**Objective:**

This investigation aims to elucidate the correlations between dietary intakes of vitamin E, B6, and niacin and the incidence of cataracts, utilizing the comprehensive NHANES 2005–2008 dataset to affirm the prophylactic roles of these nutrients against cataract formation.

**Methods:**

Using data from the NHANES 2005–2008 cycles, this analysis concentrated on 7,247 subjects after exclusion based on incomplete dietary or cataract data. The identification of cataracts was determined through participants’ self-reported ophthalmic surgical history. Nutritional intake was gauged using the automated multiple pass method, and the data were analyzed using logistic and quantile regression analyses to investigate the relationship between vitamin consumption and cataract prevalence.

**Results:**

Our analysis identified significant inverse associations between the intake of vitamins E, B6, and niacin and the risk of cataract development. Specifically, higher intakes of vitamin B6 (OR = 0.85, 95% CI = 0.76–0.96, *p* = 0.0073) and niacin (OR = 0.98, 95% CI = 0.97–1.00, *p* = 0.0067) in the top quartile were significantly associated with a reduced likelihood of cataract occurrence. Vitamin E intake showed a consistent reduction in cataract risk across different intake levels (OR = 0.96, 95% CI = 0.94–0.99, *p* = 0.0087), demonstrating a nonlinear inverse correlation.

**Conclusion:**

The outcomes indicate that elevated consumption of vitamin B6 and niacin, in conjunction with regular vitamin E intake, may have the potential to delay or prevent cataract genesis. These results suggest a novel nutritional strategy for cataract prevention and management, advocating that focused nutrient supplementation could be instrumental in preserving eye health and reducing the risk of cataracts. Further research is recommended to validate these findings and establish optimal dosages for maximum benefit.

## Highlights

• **Comprehensive analysis:** Utilizing data from the NHANES 2005–2008, this study conducted an in-depth analysis of 7,247 American adults, representing one of the largest sample sizes in research exploring the association between vitamin intake and cataract risk.
• **Key findings:** Our study demonstrates significant inverse associations between higher intakes of vitamin E, vitamin B6, and niacin and a reduced risk of developing cataracts, especially in high-intake groups. This suggests a new nutritional strategy for cataract prevention.
• **Novel insights:** We uncovered nonlinear inverse relationships between vitamin E intake and cataract risk, providing fresh perspectives on the complex interactions between vitamin consumption and eye health.
• **Practical guidance:** The findings support dietary adjustments to increase specific vitamin intakes as a potential strategy for cataract prevention and management, laying a scientific foundation for subsequent clinical trials and public health recommendations.

## Introduction

Cataracts, the loss of lens transparency, lead to symptoms like vision impairment, diminished contrast sensitivity, altered color perception, and glare (1–3). The World Health Organization reports nearly 180 million individuals globally have visual impairments, with cataracts being a significant contributor, affecting an estimated 46% of these individuals (4). Despite being largely treatable, cataracts persist as a major cause of vision loss worldwide, posing significant public health challenges across all nations (5, 6). Factors such as aging, smoking, diabetes, and ultraviolet exposure are linked to the development of age-related cataracts (7). While cataract surgery offers marked vision improvement, it is costly, with a noted shortage of surgeons in some regions (8). Addressing modifiable risks, particularly through dietary adjustments emphasizing vitamin intake, could mitigate cataracts’ health and economic impacts, as vitamins play a key role in countering oxidative stress, a fundamental trigger in cataract formation (9–11).

Vitamin E, recognized for its antioxidant properties within the ocular lens, is hypothesized to mitigate age-related cataract (ARC) development by attenuating lipid peroxidation and fortifying cellular membrane stability (12, 13). Although various epidemiological studies suggest an inverse association between vitamin E consumption and ARC incidence (14–17), this hypothesis remains contentious, with subsequent research failing to establish a consistent link (18–20).

Vitamin B6, essential for an array of metabolic, physiological, and developmental functions, functions as a critical cofactor due to its solubility in water and reactivity post-phosphorylation. Its antioxidant prowess, akin to that of carotenoids and tocopherols, plays a vital role in neutralizing oxidative stress. Notably, empirical studies have associated higher intakes of vitamin B6, along with folate and B12, with a lower prevalence of cataracts (21–25), though these findings necessitate further validation via randomized clinical trials.

Niacin, a key agent in lipid regulation, has demonstrated beneficial effects on apolipoprotein B-containing and apo A-I-containing lipoproteins, correlating with decreased nuclear cataract incidence in initial studies (26).

Given the small sample sizes in preceding research, this investigation employs data from the National Health and Nutrition Examination Survey 2005-2008 to explore more comprehensively the relationships between intake of vitamin E, B6, niacin, and cataract formation, seeking to substantiate these vitamin’ preventative roles in cataractogenesis.

## Materials and methods

### Data source and subject selection

We analyzed data from the 2005-2006 and 2007-2008 National Health and Nutrition Examination Survey (NHANES) database. This cross-sectional study aimed to assess the health and nutritional status of adults and children in the United States and is nationally representative. (27) Respondents were interviewed about their demographic, socio-economic, dietary habits, and health status. NHANES is a key project of the National Center for Health Statistics (NCHS) and is approved by the NCHS Research Ethics Review Board.

**FIGURE 1:**
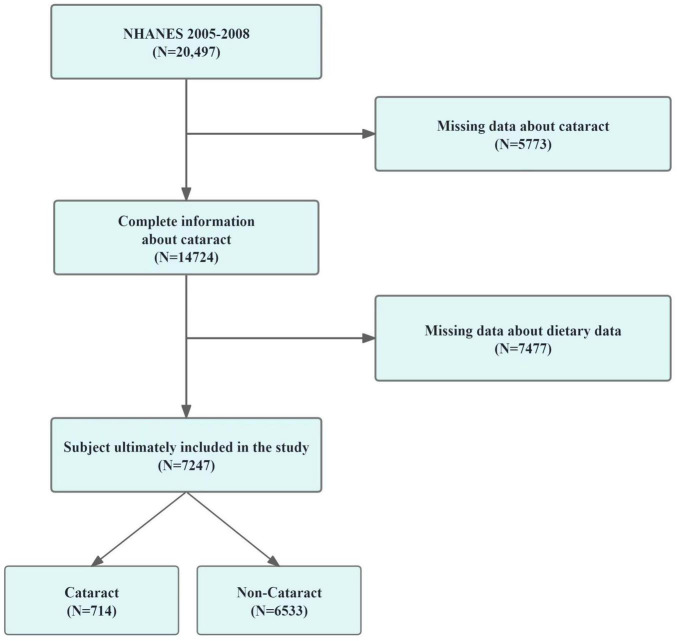
The screening process of the included studies. Flow chart of procedures from identification of eligible patients to final inclusion.

A total of 20,497 participants were involved in the survey during the 2005-2006 and 2007-2008 periods. We excluded samples missing dietary variables (*n* = 7,477) and 5,773 samples missing cataract outcomes. Therefore, the final analysis sample included 7,247 participants with complete data on diet and cataract outcomes. The methodology for participant inclusion is depicted in Figure 1.

### Cataract identification criteria

The NHANES queried adults aged 20 and older about their history of ophthalmic surgery for cataracts before their eye examination (28). Respondents confirming such surgery were identified as cataract cases in this research (VIQ071). Those with non-responses or ambiguous answers were not included. Considering the rising incidence and more lenient criteria for cataract surgery in the U.S. (29), self-reported cataract surgery is presumed to reflect clinically significant cataracts. This approach to defining cataracts has been previously utilized in other studies (30, 31).

### Determination of vitamin intake

In the face-to-face interviews, dietary information was gathered using the automated multiple pass method, a sophisticated recall methodology developed by the USDA for dietary data collection. NHANES’s Mobile Examination Center provided participants with measuring guides to accurately report their food consumption (32, 33). Dietary intake was recorded over two consecutive days, and the average of these two daily records was used to represent each participant’s dietary intake, aiming to more accurately reflect their habitual diet. It is important to note that the NHANES data does not explicitly indicate whether participants are using nutritional supplements or medications that could impact vitamin levels. The analysis incorporated all vitamin data from the NHANES 2005–2008 dataset.

### Covariates assessment

Demographic variables, including age, race/ethnicity, gender, educational attainment, and marital status, were selected as covariates in our analysis. These pieces of demographic information were collected via computer-assisted personal interviews (34). Given the link between socio-economic and living conditions and physical health, these demographic indicators were used to infer participants’ social and living situations. Additionally, diabetes mellitus, a known risk factor for cataracts (35), was incorporated as a covariate, with diabetes status determined by participants’ self-reports (36).

### Statistical analysis

Data analysis was conducted using the statistical software R (Version 4.3.2) and EmpowerStats^1^ (X&Y Solutions, Inc., Boston, MA). Given the stratified, multistage sampling methodology of NHANES, our analysis incorporated sampling weights, strata, and sampling units to reflect the survey’s complex design. Continuous variables were depicted with their means and standard errors, while categorical variables were shown with percentages and SE. Differences in demographic characteristics were assessed using chi-square or *T*-tests. Logistic regression models explored the correlation between vitamin intake and cataract incidence. Model 1 was unadjusted, Model 2 adjusted for age, race, gender, educational level, and marital status, and Model 3 additionally adjusted for diabetes mellitus. Noting significant correlations between vitamin E, B6, niacin, and cataracts, quantile regression analyses were also conducted. Forest plots were generated to illustrate the logistic regression outcomes more comprehensively. Furthermore, smoothing curve fittings examined potential nonlinear relationships between vitamin E, B6, niacin, and cataracts, considering *p*-values < 0.05 as statistically significant.

## Results

### Description of baseline information of the study sample

In our investigation based on the NHANES framework, we initially considered 20,497 participants. Following the exclusion process due to incomplete dietary or ophthalmic data, 7,247 subjects were retained for the study, while 13,250 were excluded. This selection procedure is detailed in Figure 1. Table 1 outlines demographic and characteristic data for participants, distinguishing between those with and without cataracts. Post-weighting within our cohort, the incidence of diagnosed or suspected cataracts stood at 9.85%. Notably, we found significant disparities in the intake of seven vitamins between the groups: for vitamin E, intake was 6.08 mg versus 7.02 mg (*p* < 0.001); for vitamin B1, 1.42 mg versus 1.61 mg (*p* < 0.001); for vitamin B6, 1.72 mg versus 1.98 mg (*p* < 0.001); for vitamin B12, 4.09 mg versus 5.43 mg (*p* < 0.001); for niacin, 19.59 mg versus 24.50 mg (*p* < 0.001); and total folate, 357.12 mg versus 397.96 mg (*p* < 0.001). In terms of other covariates, the analysis indicated that older individuals, females, non-Hispanic White people, less educated persons, and those with diabetes were at an increased risk of cataract formation.

**TABLE 1:** Baseline information for the study sample.

Variables	Cataract	Non-cataract	*p*-value
**Continuous variables, mean (SE)**			
Age (years)	73.98 ± 9.40	47.59 ± 16.84	<0.001
Vitamin E (mg)	6.08 ± 3.37	7.07 ± 4.43	<0.001
Retinol (mg)	0.44 ± 0.47	0.42 ± 0.48	0.443
Vitamin A (mg)	0.65 ± 0.53	0.61 ± 0.55	0.157
Vitamin B1 (mg)	1.42 ± 0.60	1.61 ± 0.80	<0.001
Vitamin B2 (mg)	1.94 ± 0.80	2.12 ± 1.08	<0.001
Niacin (mg)	19.59 ± 8.00	24.50 ± 12.07	<0.001
Vitamin B6 (mg)	1.72 ± 0.81	1.98 ± 1.09	<0.001
Total folate (mg)	357.12 ± 170.07	397.96 ± 209.12	<0.001
Vitamin B12 (mg)	4.90 ± 5.47	5.43 ± 5.85	0.023
Vitamin C (mg)	82.47 ± 63.81	90.09 ± 81.25	0.015
Vitamin K (mg)	95.67 ± 111.08	97.35 ± 116.90	0.714
**Category variables, (%)**			
Gender (*n*, %)			0.165
Male	324 (45.38%)	3143 (48.11%)	
Female	390 (54.62%)	3390 (51.89%)	
Race (*n*, %)			<0.001
Mexican	53 (7.42%)	1243 (19.03%)	
Other Hispanic	50 (7.00%)	534 (8.17%)	
Non-Hispanic white	488 (68.35%)	3101 (47.47%)	
Non-Hispanic black	106 (14.85%)	1416 (21.67%)	
Other race	17 (2.38%)	239 (3.66%)	
Education (*n*, %)			<0.001
Less than 9th grade	144 (20.17%)	703 (10.77%)	
9–11Th grade (includes 12th grade with no diploma)	111 (15.55%)	1074 (16.45%)	
High school grad/GED or equivalent	193 (27.03%)	1591 (24.37%)	
Some college or aa degree	154 (21.57%)	1851 (28.35%)	
College graduate or above	112 (15.69%)	1310 (20.06%)	
Marital Status (*n*, %)			<0.001
Married or living with partner	381 (53.36%)	4127 (63.20%)	
Unmarried or other	333 (46.64%)	2403 (36.80%)	
Diabetes mellitus (*n*, %)			<0.001
Yes	190 (27.38%)	690 (10.73%)	
No	504 (72.62%)	5740 (89.27%)	

### Association between the intake of seven vitamins and the presence of cataracts

**FIGURE 2:**
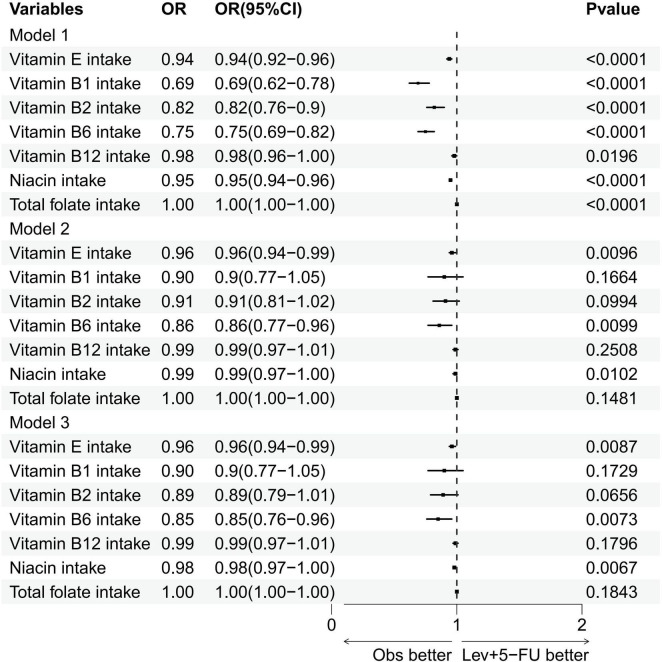
Forest plot of logistic regression results. Multivariate logistic regression analysis of the association between various vitamin intakes and the specified outcome. OR, odds ratios; CI, confidence intervals.

**FIGURE 3:**
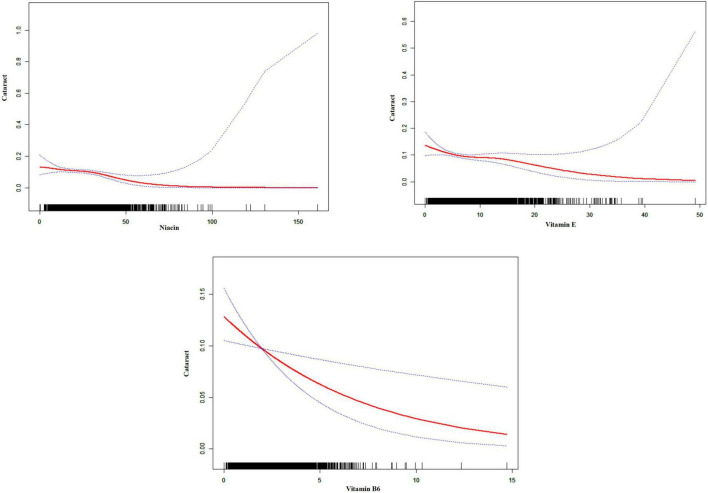
Smoothed curve fitting plot. The charts indicate a potential non-linear association between vitamin intake and cataract prevalence. The red line represents the fitted curve, and the blue dotted lines represent the 95% confidence intervals. The black vertical lines at the bottom of each chart indicate the distribution of vitamin intake.

Figure 2 and Table 2 detail the relationships between the consumption of vitamin E, B1, B2, B6, B12, niacin, and total folate and the prevalence of cataracts, as established through multivariate logistic regression analyses. The findings across all models reveal significant negative associations for vitamin E intake (model 1: OR = 0.94, 95% CI = 0.92–0.96; model 2: OR = 0.96, 95% CI = 0.94–0.99; model 3: OR = 0.96, 95% CI = 0.94–0.99), vitamin B6 (model 1: OR = 0.75, 95% CI = 0.69–0.82; model 2: OR = 0.86, 95% CI = 0.77–0.96; model 3: OR = 0.85, 95% CI = 0.76–0.96), and niacin (model 1: OR = 0.95, 95% CI = 0.94–0.96; model 2: OR = 0.99, 95% CI = 0.97–1.00; model 3: OR = 0.98, 95% CI = 0.97–1.00) regarding cataract development. Additionally, smoothing curve fitting analyses substantiated the nonlinear inverse relations between the intakes of vitamin E, B6, niacin, and cataract risks, as depicted in Figure 3.

**TABLE 2:** Association between intake of vitamin E, vitamin B1, vitamin B2, vitamin B6, vitamin 12, niacin, total folate, and cataract.

Variables	Model 1^a^ OR (95%CI)	*p*-value	Model 2^b^ OR (95%CI)	*p*-value	Model 3^c^ OR (95%Cl)	*p*-value
Vitamin E intake	0.94 (0.92–0.96)	<0.0001	0.96 (0.94–0.99)	0.0096	0.96 (0.94–0.99)	0.0087
Vitamin B1 intake	0.69 (0.62–0.78)	<0.0001	0.9 (0.77–1.05)	0.1664	0.9(0.77–1.05)	0.1729
Vitamin B2 intake	0.82 (0.76–0.9)	<0.0001	0.91 (0.81–1.02)	0.0994	0.89 (0.79–1.01)	0.0656
Vitamin B6 intake	0.75 (0.69–0.82)	<0.0001	0.86 (0.77–0.96)	0.0099	0.85 (0.76–0.96)	0.0073
Vitamin B12 intake	0.98 (0.96–1.00)	0.0196	0.99 (0.97–1.01)	0.2508	0.99 (0.97–1.01)	0.1796
Niacin intake	0.95 (0.94–0.96)	<0.0001	0.99 (0.97–1.00)	0.0102	0.98 (0.97–1.00)	0.0067
Total folate intake	1.00 (1.00–1.00)	<0.0001	1.00 (1.00–1.00)	0.1481	1.00 (1.00–1.00)	0.1843

^a^Model 1: a no adjusted.

^b^Model 2: adjusted for age, race, gender, educational level and marital status.

^c^Model 3: further adjusted for diabetes mellitus.

### Relationship of different quartiles of vitamin E, B6 and niacin with the presence of cataract

**FIGURE 4:**
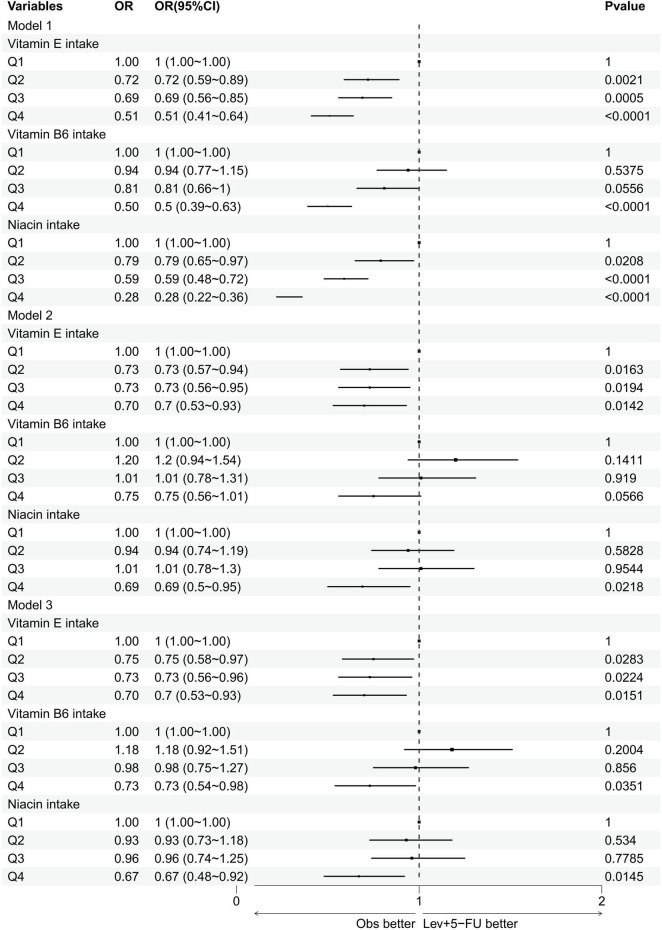
Forest plot of logistic regression results. This figure presents the results of multivariate logistic regression analyses evaluating the association between the intake of vitamin E, vitamin B6, and niacin (divided into quartiles Q1–Q4) and the incidence of cataracts. OR, odds ratios; CI, confidence intervals.

Figure 4 and Table 3 explore the relationship between varying levels of vitamin E, B6, and niacin intake, segmented into quartiles, and cataract incidence. For the initial three quartiles—Q1 representing low vitamin intake, Q2 indicating normal intake, and Q3 denoting moderate to high intake—no substantial links to cataract prevalence were detected for vitamin B6 and niacin. However, within the highest intake group (Q4), significant inverse associations with cataract occurrence were identified for both vitamin B6 and niacin across all models (vitamin B6: model 1: OR = 0.5, 95% CI = 0.39–0.63; model 2: OR = 0.75, 95% CI = 0.56–1.01; model 3: OR = 0.73, 95% CI = 0.54–0.98; niacin: model 1: OR = 0.28, 95% CI = 0.22–0.36; model 2: OR = 0.69, 95% CI = 0.5–0.95; model 3: OR = 0.67, 95% CI = 0.48–0.92). Additionally, a significant inverse correlation between vitamin E intake and cataract risk was consistently observed across Q2, Q3, and Q4 quartiles in all models.

**TABLE 3:** Association between vitamin E, vitamin B6, niacin intake levels, and cataract in different quartiles.

Variables		Model 1^a^ OR (95%CI)	*p*-value	Model 2^b^ OR (95%CI)	*p*-value	Model 3^c^OR (95%Cl)	*p*-value
Vitamin E intake	Q1	1 (1.00–1.00)	1	1 (1.00–1.00)	1	1 (1.00–1.00)	1
	Q2	0.72 (0.59–0.89)	0.0021	0.73 (0.57–0.94)	0.0163	0.75 (0.58–0.97)	0.0283
	Q3	0.69 (0.56–0.85)	0.0005	0.73 (0.56–0.95)	0.0194	0.73 (0.56–0.96)	0.0224
	Q4	0.51 (0.41–0.64)	<0.0001	0.7 (0.53–0.93)	0.0142	0.7 (0.53–0.93)	0.0151
Vitamin B6 intake	Q1	1 (1.00–1.00)	1	1 (1.00–1.00)	1	1 (1.00–1.00)	1
	Q2	0.94 (0.77–1.15)	0.5375	1.2 (0.94–1.54)	0.1411	1.18 (0.92–1.51)	0.2004
	Q3	0.81 (0.66–1)	0.0556	1.01 (0.78–1.31)	0.919	0.98 (0.75–1.27)	0.856
	Q4	0.5 (0.39–0.63)	<0.0001	0.75 (0.56–1.01)	0.0566	0.73 (0.54–0.98)	0.0351
Niacin intake	Q1	1 (1.00–1.00)	1	1 (1.00–1.00)	1	1 (1.00–1.00)	1
	Q2	0.79 (0.65–0.97)	0.0208	0.94 (0.74–1.19)	0.5828	0.93 (0.73–1.18)	0.534
	Q3	0.59 (0.48–0.72)	<0.0001	1.01 (0.78–1.3)	0.9544	0.96 (0.74–1.25)	0.7785
	Q4	0.28 (0.22–0.36)	<0.0001	0.69 (0.5–0.95)	0.0218	0.67 (0.48–0.92)	0.0145

^a^Model 1: no adjusted.

^b^Model 2: adjusted for age, race, gender, educational level and marital status.

^c^Model 3: further adjusted for diabetes mellitus.

## Discussion

The 2019 World Health Organization’s World Report on Vision confirms that over a billion people worldwide suffer from vision impairments that are preventable or treatable to avoid blindness. Additionally, the number of people experiencing partial or severe blindness is increasing at an alarming rate. Cataracts and refractive errors account for half of the cases of blindness or severe vision impairment. Although surgical treatments exist for cataracts, many eye diseases leading to blindness, including age-related macular degeneration (AMD), remain incurable. This highlights the importance of researching the mechanisms behind these diseases and slowing their progression through preventive measures. Diet and lifestyle are two of the most critically studied factors, yet they remain poorly understood by patients. These factors seem to significantly influence both the onset and progression rate of these diseases (37).

In this study, we utilized the NHANES database to investigate the potential association between the intake of vitamin E, B6, and niacin and the occurrence of cataracts.

Our findings indicate no significant correlation between the normal or low intake levels of vitamin B6 and niacin and cataract development. However, a significant association was observed with high intakes of these vitamin. Conversely, a regular intake of vitamin E showed a notable correlation with the incidence of cataracts.

In 1922, Evans and Bishop (38) unveiled Vitamin E, which encompasses diverse fat-soluble compounds (38). This category includes alpha, beta, gamma, and delta types of both tocopherol and tocotrienol, originating from plants via homogentisic acid synthesis. Serum, red blood cells, and various ocular tissues mainly contain the alpha and gamma-tocopherol forms, with alpha-tocopherol being predominant. In comparison, beta and delta tocopherols are less present in plasma (39, 40). Noteworthy is alpha-tocopherol’s significant presence in mitochondria and endoplasmic reticulum membranes, areas known for intense free-radical activity (40). Vitamin E is essential in mitigating oxidative stress, protecting cell membranes, influencing platelet aggregation, and stimulating protein kinase C (40). The ocular lens, composed of 63% water and 35% proteins (41), can turn opaque and lead to cataracts under various stress conditions, underscoring these vitamins’ importance in eye health. Various factors, including alterations in water content, abnormal protein clustering, and exposure to detrimental elements like UV and X-ray radiation, steroids, certain chemicals, or drugs, and the effects of systemic or local ailments such as diabetes, smoking, and poor nutrition, can induce lens opacity, heightening cataract risks. Such risks increase with age and are more prevalent in women than men (42). Lens damage is often exacerbated by xenobiotics that induce free radicals, leading to detrimental effects like membrane lipid peroxidation, protein inactivation, and aggregation, eventually causing lens opacification. Such free radicals particularly target the polyunsaturated fatty acids within the lens, catalyzing cataract formation. Vitamin E is known to counteract lipid peroxidation, thus aiding in the preservation of membrane integrity and functionality (43). Research has demonstrated that vitamin E can mitigate the development of cataracts induced by substances such as galactose and aminothiazole in rabbits and protect against the photooxidation of lens lipids (44). Moreover, similar to vitamin C, vitamin E has shown potential efficacy against age-related and irradiation-induced cataracts (45–47), with some studies proposing its clinical application for cataract prevention (48). Its role as a free radical scavenger, attributed to its lipid solubility and antioxidant characteristics, is believed to be pivotal in maintaining membrane health. For instance, a study by Trevithick et al. illustrated how rat lenses incubated in a solution with high glucose and serum levels exhibited increased opacity and globular degeneration over time, which was mitigated by the presence of vitamin E, highlighting its protective effects on cellular structures. In the studied lenses, the concentrations of glucose, sorbitol, and fructose exceeded those in the controls. When vitamin E at a concentration of 2.4 μm was introduced, lens swelling subsided, and normal volume was restored more quickly, despite uniform osmolarity across all external media (49). Vitamin E demonstrated a protective effect on cell membranes by influencing their permeability and osmotic balance. Ohta et al. (50) observed that cataracts developed in lenses from male Wistar rats following incubation with methylprednisolone (1.5 mg/ml). The addition of vitamin E to the incubation medium inhibited further progression of cataracts, reduced increases in lipid peroxide levels and the Na^+^/K^+^ ratio, and ameliorated decreases in reduced glutathione, glyceraldehyde-3-phosphate dehydrogenase, and Na^+^/K^+^-ATPase activities (50). Vitamin E thereby mitigated steroid-induced cataractogenesis by safeguarding the lenses from oxidative harm and functional decline of their membranes. However, extensive experimental studies and randomized clinical trials have consistently indicated that vitamin E does not confer cataract protection in humans (51, 52).

Research emphasizing the ineffectiveness of vitamin E against human cataracts, even when administered at 400 IU three times weekly (53) or daily (52), underscores the challenge of identifying an optimal dosage. This concern extends beyond just the total vitamin E intake to the specific amounts of its components.

Therefore, advancing research into the different vitamin E variants is crucial to ascertain their potential benefits, understand the optimal dosages for each, and explore their prospective roles in cataract prevention and broader clinical applications as a significant and powerful antioxidant (10). Moreover, studies have shown that vitamins and antioxidants like vitamin C, vitamin E, and carotenoids may influence the pathogenesis of diabetic retinopathy (DR) by reducing retinal neovascularization, restoring blood flow, and protecting against free radicals (54). Additionally, vitamins C and E seem to inhibit the production of vascular endothelial growth factor (VEGF) in animal models and decrease the accumulation of advanced glycation end products (AGEs). Thus, the antioxidant effects of vitamin E are critically important and widely recognized in the development and progression of certain ocular diseases.

Vitamin B6, known for its broad involvement in metabolic, physiological, and developmental functions, stands out due to its water solubility and reactivity upon phosphorylation, making it an essential cofactor in numerous biochemical reactions. It is also recognized for its potent antioxidant capacity, comparable to that of carotenoids and tocopherols, effectively neutralizing reactive oxygen species (55). Vitamin B6 exists in multiple forms, with pyridoxal 5′-phosphate (P5P) being the safest and most effective natural active form for reducing homocysteine levels. Elevated homocysteine levels disrupt autophagy, leading to abnormal crystallin protein expression, a key factor in cataract development (56). We hypothesize that vitamin B6 may reduce cataract risk by eliminating homocysteine, thus providing a potential protective mechanism. Studies have demonstrated that vitamin B6 can mitigate protein oxidation and cloudiness in cataract models treated with mercury, suggesting its potential role in delaying or even preventing cataract progression, particularly in diabetic individuals (57). The antioxidant action of vitamin B6, specifically its ability to scavenge oxygen free radicals in the lens, was significant in our findings, where high doses (Q4) correlated with a reduced incidence of cataracts, warranting further investigation into optimal dosages.

Niacin, or vitamin B3, plays a crucial role in converting to nicotinamide adenine nucleotide, crucial for energy extraction from nutrients and maintaining genetic stability. It can be synthesized from dietary tryptophan at a ratio of 60 mg to 1 mg of niacin (58). A case report has observed the occurrence of cataracts in patients with pellagra, enhancing our understanding of the relationship between Vitamin B3 deficiency and cataract development. Vitamin B3 is crucial for the synthesis of glutathione in the lens, where glutathione acts as a primary antioxidant against oxidative damage. In this particular case, the patient also had beta-thalassemia, a condition that increases iron load and oxidative stress, accelerating cataract formation (59). This finding emphasizes the importance of maintaining adequate Vitamin B3 levels to prevent oxidative stress-induced ocular diseases. Additionally, for individuals predisposed to genetic conditions, proper nutrition and antioxidant defense are vital for maintaining eye health. While high doses of niacin have been linked to lowered serum cholesterol and triglycerides and a decreased risk of myocardial infarction, they may also elevate the likelihood of cardiac arrhythmias. Our study noted a significant association between high niacin intake (Q4) and reduced cataract incidence, highlighting the necessity for further research to delineate the appropriate dosage, considering potential adverse effects.

This study’s strengths lie in its focus on the intake of vitamin E, B6, and niacin and their relation to cataract occurrence, supported by a considerable sample size. However, there are limitations: A significant limitation of our study is the reliance on participants’ self-reported history of eye surgeries to determine the presence of cataracts. While this approach offers a practical method for collecting data across large populations, it inherently carries accuracy risks. Using self-reported history of ophthalmic surgery as an indicator for cataract presence might be a potential limitation, as self-reported data can be less accurate than clinical diagnoses. This issue could impact the reliability of the observed association between vitamin intake and cataract occurrence, as the data may not accurately reflect the true incidence of clinically significant cataracts. To address this limitation in future studies, more objective diagnostic criteria or verification through medical records may be necessary to enhance the accuracy of cataract identification. Additionally, NHANES ophthalmic data does not specify the type of cataracts or provide detailed correlations between vitamin intake and various forms of cataracts. Moreover, relying on self-reported dietary information may introduce inaccuracies, and the NHANES database does not explicitly indicate whether participants are using nutritional supplements or medications that could affect vitamin metabolism. This limitation may impact our understanding of the relationship between vitamin intake and cataract occurrence. Finally, the inherent bias of cross-sectional studies precludes establishing causality.

Although this study shows a correlation between a high intake of vitamins E, B6, and niacin and a reduced risk of cataracts, caution is warranted when applying these findings to clinical practice and daily life. Firstly, the results of this study are based on cross-sectional data, which cannot be used to infer causality directly. Furthermore, while the statistical analysis shows a significant negative correlation, this does not imply that increasing the intake of these vitamins can be directly employed for the prevention or treatment of cataracts. In fact, excessive intake of certain vitamins may present potential health risks.

Before applying these research findings, further studies are necessary to validate these relationships and explore the optimal dosages and supplementation methods for these vitamins. For instance, future research could involve designing randomized controlled trials to test the effects of specific vitamin doses on cataract development and to investigate the potential impacts of long-term supplementation on eye health.

## Conclusion

Vitamin E, combined with high doses of vitamin B6 and niacin, shows promise in potentially delaying or preventing cataract development. Such findings introduce a novel dietary approach for the clinical prevention and management of cataracts, suggesting that targeted nutrient supplementation could play a key role in maintaining ocular health and mitigating the risk of cataract formation. This insight paves the way for further research to confirm these relationships and to determine the optimal dosages for therapeutic efficacy while ensuring safety and minimizing adverse effects.

## Data availability statement

The original contributions presented in this study are included in the article/supplementary material, further inquiries can be directed to the corresponding author.

## Author contributions

G-BZ: Conceptualization, Data curation, Formal analysis, Investigation, Methodology, Project administration, Resources, Software, Supervision, Validation, Writing – original draft, Writing – review and editing. XL: Data curation, Methodology, Project administration, Validation, Writing – review and editing. S-NW: Data curation, Investigation, Methodology, Software, Writing – review and editing. S-QZ: Conceptualization, Investigation, Supervision, Writing – review and editing. Z-JZ: Formal analysis, Methodology, Validation, Writing – review and editing. ND: Conceptualization, Data curation, Formal analysis, Funding acquisition, Investigation, Methodology, Project administration, Resources, Software, Supervision, Validation, Visualization, Writing – review and editing.

## Footnotes

1
http://www.empowerstats.com
